# Deciphering the H-Bonding Preference on Nucleoside Molecular Recognition through Model Copper(II) Compounds

**DOI:** 10.3390/ph14030244

**Published:** 2021-03-09

**Authors:** Inmaculada Velo-Gala, Miquel Barceló-Oliver, Diego M. Gil, Josefa M. González-Pérez, Alfonso Castiñeiras, Alicia Domínguez-Martín

**Affiliations:** 1 Faculdade de Engenharia da Universidade do Porto, Rua Dr. Roberto Frias, 4200-465 Porto, Portugal; invega@fe.up.pt; 2 Department of Chemistry, Universitat de les Illes Balears, Crta de Valldemossa km 7.5, 07122 Palma de Mallorca, Spain; miquel.barcelo@uib.es; 3 Instituto de Biotecnología Farmaceútica y Alimentaria, Consejo Nacional de Investigaciones Científicas y Técnicas—Universidad Nacional de Tucumán, Instituto de Química Orgánica, Facultad de Bioquímica, Química y Farmacia, Universidad Nacional de Tucumán, Ayacucho 471, San Miguel de Tucumán T4000INI, Argentina; dmgil@fbqf.unt.edu.ar; 4 Department of Inorganic Chemistry, Faculty of Pharmacy, University of Granada, 18071 Granada, Spain; jmgp@ugr.es; 5 Department of Inorganic Chemistry, Faculty of Pharmacy, University of Santiago de Compostela, 15782 Santiago de Compostela, Spain; alfonso.castineiras@usc.es

**Keywords:** acyclovir, molecular recognition, DFT, non-covalent interactions, H-bonds

## Abstract

The synthetic nucleoside acyclovir is considered an outstanding model of the natural nucleoside guanosine. With the purpose of deepening on the influence and nature of non-covalent interactions regarding molecular recognition patterns, three novel Cu(II) complexes, involving acyclovir (acv) and the ligand receptor N-(2-hydroxyethyl)ethylenediamine (hen), have been synthesized and thoroughly characterized. The three novel compounds introduce none, one or two acyclovir molecules, respectively. Molecular recognition has been evaluated using single crystal X-ray diffraction. Furthermore, theoretical calculations and other physical methods such as thermogravimetric analysis, infrared and UV-Vis spectroscopy, electron paramagnetic resonance and magnetic measurements have been used. Theoretical calculations are in line with experimental results, supporting the relevance of the [metal-N7(acv) + H-bond] molecular recognition pattern. It was also shown that (hen)O-H group is used as preferred H-donor when it is found within the basal coordination plane, since the higher polarity of the terminal (hen)O-H versus the N-H group favours its implication. Otherwise, when (hen)O-H occupies the distal coordination site, (hen)N-H groups can take over.

## 1. Introduction

Synthetic nucleoside analogues have been widely used as suitable models for the study of nucleic acids and its applications along different research fields [1,2,3,4,5]. In contrast to the furanose ring of natural nucleosides, which undergoes opening-cyclization equilibrium, the presence of an acyclic side-chain in some synthetic nucleosides offers a remarkable chemical stability. This fact can be conveniently harnessed by structural studies, especially those involving metal binding and ligand interaction. In this context, the similarities between acyclic and natural nucleosides are worth noting, despite the different chemical nature of the O-ether group and the furanosic oxygen in acyclic and natural nucleosides, respectively.

**Scheme 1:**
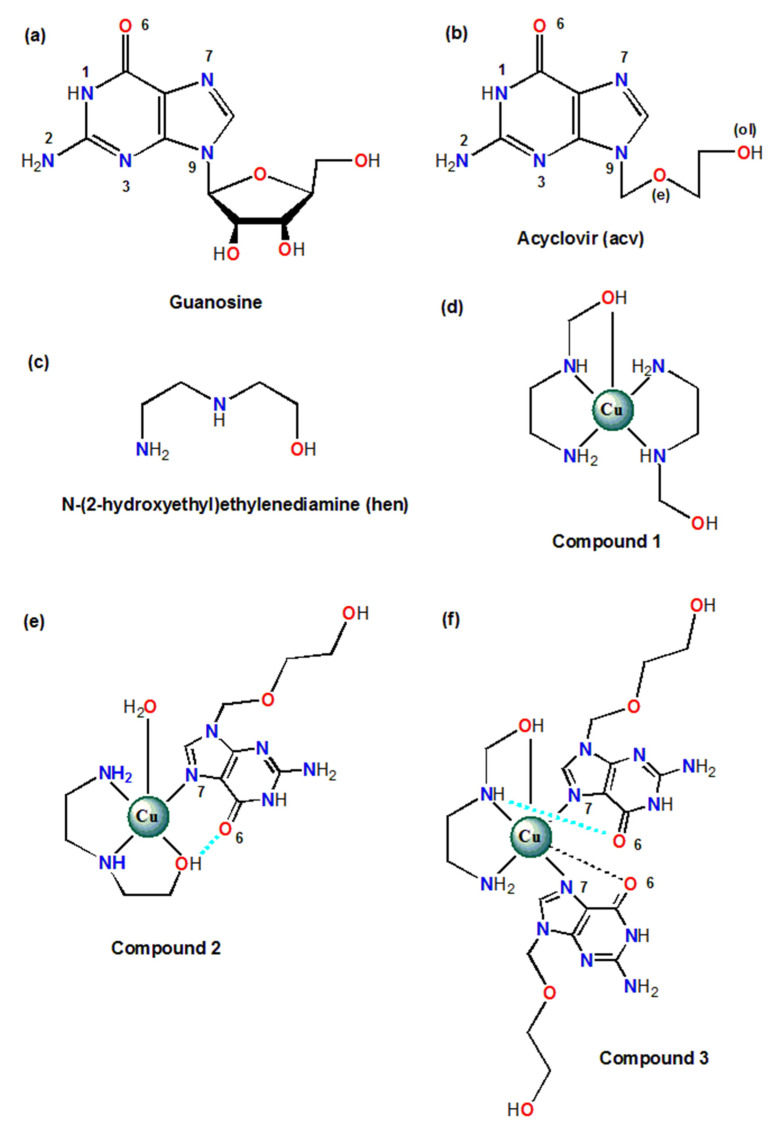
Formula of (**a**) guanosine and (**b**) acyclovir, 9-(2-hydroxyethoxymethyl)guanine (acv), with conventional notation of purines; and (**c**) the chelating ligand N-(2-hydroxyethyl)ethylenediamine (hen). In addition, schematic view of compounds **1**–**3** (**d**–**f**) are provided noting the acv numbering. H-bonds are depicted with cyan dashed lines. For clarity reasons, only the 4 + 1 geometry has been represented in (**d**). The black dashed line in (**f**) represents a Cu-O interaction present in compound **3**.

Several acyclic nucleoside derivatives have proved to have antiviral activity [6]. For instance, acyclovir (acv) is a representative example of an acyclic synthetic nucleoside (Scheme 1) prescribed worldwide as an antiviral agent against *Herpesviridae* viruses. In vivo, acyclovir is phosphorylated by viral thymidine kinase into acyclovir triphosphate, which competes with deoxyguanosine triphosphate, and competitively inhibits viral DNA polymerase [7].

Acyclovir is also considered an outstanding structural model of endogenous guanosine (Scheme 1). Because of their similarities, the interaction of this synthetic nucleoside with metal ions present in biological systems is worth studying. Along the past decades, mainly three research groups have contributed to enlighten the metal binding abilities of acyclovir [8,9,10,11,12,13,14,15,16,17,18,19,20,21,22]. According to this information, acv preferentially acts as monodentate ligand, involving the purine moiety, with the M-N7(acv) coordination bond acting in cooperation with an intramolecular interligand D-H···O6(acv) interaction (D = oxygen or nitrogen H-donor atoms) [8,10,11,13,16,18,19,20,21,22]. Besides this preferred molecular recognition pattern, further studies have reported more unconventional modes for acv such as the N7,O6-chelating mode or even the tetradentate μ_3_-N7,O6,O(e),O(ol) mode [12,14,17].

Besides metal binding, non-covalent interactions are also believed to play a key role in biological processes. For instance, they are essential to build the three-dimensional structure of large biomolecules such as nucleic acids or even induce specific, although transient, binding between biomolecules [23]. A previous study from our group was devoted to rationalize the cooperation between the coordination bond Cu(II)-N7(acv) and interligand H-bonds [10]. To this purpose, two amino acids (i.e., glycyglycinate(2-) and iminodiacetate(2-)) were chosen according to their suitability or not to form such H-bonding interactions relying on the presence of appropriate terminal H-donor groups.

In this work, a step further is proposed, wondering about the preference for the nature of H-donor groups in molecular recognition patterns. The ligand N-(2-hydroxyethyl)ethylenediamine (hen, Scheme 1) offers two terminal H-donor groups, i.e., –OH and –NH_2_ groups, which are able to actively participate in H-bonding interactions. Moreover, hen is a versatile ligand that is able to play either a N,N’-bidentate role, similar to that of ethylenediamine, or a N,N’,O-tridentate role. Additional flexibility can be introduced in the system by using copper(II) as metal centre, since the Jahn-Teller effect associated to the 3d^9^ electronic configuration of Cu(II) ions allows featuring different geometries and driving the hen chelator to different conformations. For instance, a crystallographic review in the Cambridge Crystallographic Database (CSD) on metal complexes involving the chelating ligand hen reveals that, regarding the tridentate fashion, both mer-NO_2_ and fac-N_2_+O(distal) conformations can be adopted, with the latter being the most frequent. In particular, 2 examples have been found for Cu(II)-hen complexes in mer-NO_2_ conformation, ref. codes BHEDCB or HANFOR and HANFIL, while 14 examples have been found for Cu(II)-hen complexes in fac-N_2_+O(distal) conformation, most of them as bis-chelate compounds, including ref. codes such as AYIQEF, DAYKUM or GOPYAL.

## 2. Results and Discussion

### 2.1. Analysis of the Structural and Theoretical Studies of Compound [Cu(hen)_2_]SO_4_ (***1***)

**Figure 1:**
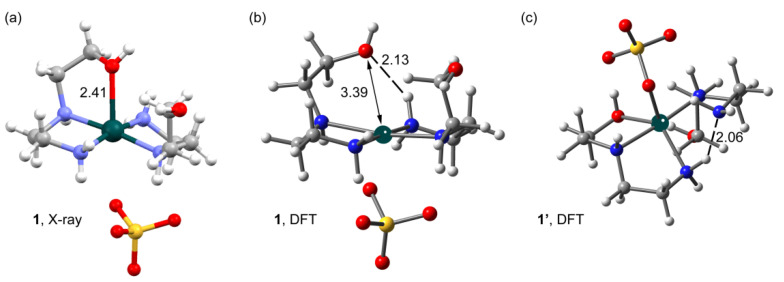
(**a**) X-ray structure of compound **1**; (**b**) B3LYP-D3/def2-SVP optimized geometry of **1**; (**c**) B3LYP-D3/def2SVP optimized geometry of **1**′ (see text). Distances are given in Å. H-Bonds depicted in dotted cyan lines. Atoms colour code: carbon—grey; hydrogen—white; nitrogen—blue; oxygen—red; copper—dark green; sulfur—yellow.

**Figure 2:**
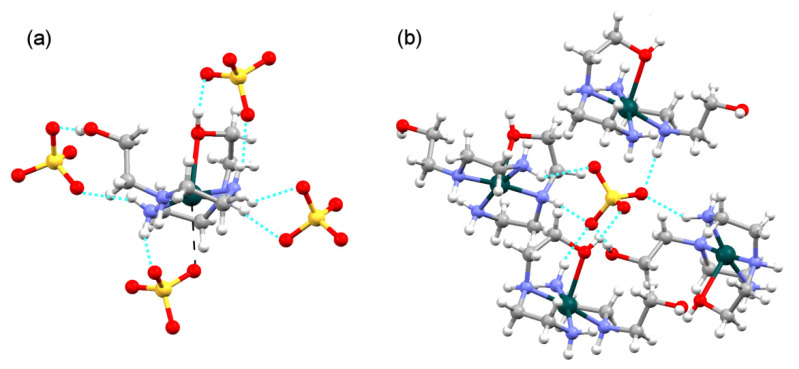
H-bonding cation-anion recognition in the crystal of compound **1**. H-bonds depicted as dotted cyan lines. (**a**) Complex cation surrounded by four sulfate anions. The Cu-O(sulfate) contact (2.879(3) Å) is depicted as dashed black; (**b**) Sulfate anion surrounded by four complex cations. Atoms colour code: carbon—grey; hydrogen—white; nitrogen—blue; oxygen—red; copper—dark green; sulfur—yellow.

The asymmetric unit of compound **1** consists of the bis-chelate [Cu(hen)_2_]^2+^ cation and one sulfate counter anion. The complex cation shows an elongated octahedral environment, type 4 + 1 + 1 *, with four nearly coplanar Cu-(N or O) donors about ~2 Å and other two Cu-O distal coordination bonds. In this compound, * indicates that one of the distal interatomic bonds has a rather large distance (Cu1-O24(sulfate) 2.879(3) Å), fairly close to the sum of Van der Waals radii [Cu (1.40 Å) + O (1.52 Å) = 2.92 Å]. The extreme weakness of this latter distal bond indeed explains some unexpected spectral behaviour of this compound, since the referred distal position can be easily lost. In fact, it could also be well considered a mere contact. For instance, if only a 4 + 1 polyhedron is considered, i.e., if the Cu-O(sulfate) contact is obviated, the calculation of the Addison-Reedijk parameter (τ = θ − φ/60 = 0.08) would reveal an almost regular square-based pyramidal geometry (Figure 1a). On the other hand, in the crystal, the largest Cu-O(sulfate) contact is assisted by the rather linear anion-cation H-bond N11-H11-O25 (2.90(1) Å, 172.0°). If such interaction is considered, one might argue a certain ‘molecular nature’ of the ‘ion pair’ constituents in **1** (Figure 2).

It is interesting to note that each of the hen chelators features different denticities. While one hen ligand displays a tridentate fac-N_2_+O(distal) conformation, the other acts as a N,N-bidentate chelator (Scheme 1d). It seems clear that the different denticity observed for hen in compound **1** (i.e., bidentate and tridentate mode) is related to the coordinating behaviour of the sulfate anion. Hence, the copper(II) coordination polyhedron is defined by four nearly coplanar Cu-N bonds plus one distal Cu-O(ol) bond and the above-referred weak Cu-O(sulfate) contact (Table S1.1).

Powder electron paramagnetic resonance (EPR) spectra of this compound show the characteristics of a rhombic g tensor at both X and Q-bands (Figure S4.1). Interestingly, the calculated *g* values (<*g*> = 2.111) do not agree with a simpler 4 + 1 copper(II) coordination. This fact and the absence of hyperfine lines indicate that the *g* tensor deduced from the experimental spectra is not the molecular one but the exchange tensor. Interactions through sulfate groups between magnetically non-equivalent Cu(II) ions at a distance of 6.5 Å are weak but large enough to average their individual resonances. The exchange interaction parameter G is 2.53 which, according to Hathaway [24], implies non negligible exchange interaction between Cu(II) ions. For this reason, the observed spectrum does not perfectly reflect the geometry of the environment of the Cu(II) ions. In any case, the experimental *g* is appreciably higher than the free electron value (*g*_0_ = 2.0023) indicating a dx^2^ − y^2^ ground state, as correspond to axially elongated octahedral or square pyramidal environments for Cu(II) ions.

In addition, a DFT study was performed to analyze the possible competition between the O- and N-donors in the hen chelator in compounds **1** and **2**. Figure 1 shows the X-ray structure of **1**, considering a 4 + 1 coordination polyhedron, and two DFT optimized structures. While Figure 1b is the result of the optimization using the X-ray coordinates as starting point, the other DFT optimized structure (Figure 1c) is a hypothetical one where the NH_2_ group, instead of the OH group, is placed in the distal coordination site as starting point (denoted as 1′). It can be observed that in the absence of neighbouring molecules, inherent to DFT calculations, compound **1** still presents a similar geometry to the experimental one (Figure 1a). The main difference is found in the distal Cu···O bond, which is broken in the optimized isolated molecule, and a new N–H···O H-bond is formed instead (Figure 1a vs. Figure 1b). It is remarkable that when the NH_2_ group is placed in the axial position, instead of the OH group, the final geometry is completely different (Figure 1c). Herein the O-sulfate occupies the distal position and the (hen)OH group replaces one amino group in the basal plane, leaving one uncoordinated hen amino group that is involved in a H-bond with an adjacent hen ligand. This result agrees well with the information gathered in a thorough structural analysis within the CSD that shows the preference of the OH group to occupy the distal position in [Cu(hen)_2_]^2+^ octahedral complexes [25,26,27]. Interestingly, DFT optimized structures 1 and 1′ are very close in energy, with 1′ (the one with the sulfate anion coordinated in the distal coordination site) being 5 kcal/mol more stable than 1. Nonetheless, unlike compound **1**, in these reported complexes both hen ligands exhibited the same fac-N_2_+O(distal) tridentate conformation (Figure 1).

In compound **1**, cation and anion recognize each other by two intermolecular H-bonding interactions involving the N-H(hen) as H-donors and O(sulfate) as acceptor (Table S1.2). In addition, the crystal of compound **1** shows each cation surrounded by four sulfate anions, connected by H-bonding interactions, where all N-H and O-H bonds of both non-equivalent hen ligands are involved as H-donors for appropriate O-sulfate acceptors (Figure 2a). Analogously, each sulfate anion is H-bonded to four complex cations (Figure 2b). Indeed, it is the aggregate strength of multiple H-bonds connecting complex cations with anions which helps to stabilize the three-dimensional structure of the crystal.

### 2.2. Analysis of the Structural and Theoretical Studies of Compound [Cu(hen)(acv)(H_2_O)](NO_3_)_2_ (***2***)

**Figure 3:**
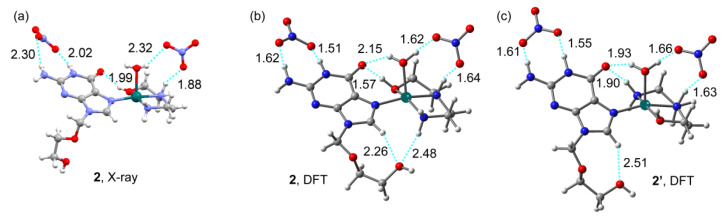
(**a**) X-ray structure of compound **2**; (**b**) B3LYP-D3/def2-SVP optimized geometry of **2**; (**c**) B3LYP-D3/def2-SVP optimized geometry of **2**′ (see text). H-bonds are depicted in black dashed lines. Distances in Å. Atoms colour code: carbon—grey; hydrogen—white; nitrogen—blue; oxygen—red; copper—dark green.

The asymmetric unit of compound **2** consists of a complex cation and two non-coordinating nitrate counter anions. In this occasion, one acv ligand is introduced in the complex cation, bound to the metal center. The copper(II) centre features a square-based pyramidal geometry, type 4 + 1, where the four closest donors are provided by the chelator hen in mer-NO_2_ conformation and the N7 donor of the guanosine moiety of acv (Cu-N27(acv) 2.010(5) Å, Scheme 1e, Table S2.1). The distal position is satisfied by one aqua ligand, which is responsible for the slight shift of the Cu(II) ion out of the basal coordination plane (P = 0.19 Å). Regarding the molecular recognition pattern, the orientation of the –OH group in hen allows the cooperation of the Cu-N27 bond with an intramolecular interligand interaction type (hen)O-H···O6(acv), thus involving the acyclovir O6-carbonyl group as H-acceptor (Table S2.2, Figure 3a).

Powder EPR spectra of this compound unambiguously indicate isolated Cu(II) ions in axially distorted sites (Figure S4.2). The hyperfine structure, resulting from the interaction between the nuclear spin of copper isotopes (^63^Cu and ^65^Cu, I = 3/2) and the unpaired d^9^ electrons (S = 1/2), is well resolved in the low field region of the spectra and allows an accurate determination of the parallel Spin Hamiltonian parameters: *g*_‖_ = 2.235 and A_‖_ = 185 Gauss (193 × 10^−4^ cm^−1^). The hyperfine splitting is not resolved in the high field part of the spectra resulting in an intense line with *g*_⊥_ = 2.058. The calculated *g* and A values are indicative of a dx^2^ − y^2^ ground state, in good agreement with the distorted 4 + 1 pyramidal geometry described above for this complex. The covalent nature of the in-plane σ-bonding has been evaluated by calculating the α^2^ value from the Spin Hamiltonian parameters [28]. α^2^ is close to 1 for a completely ionic bond between the metal and the ligands and decreases with increasing covalent character. The calculated value of 0.833 indicates a notable covalency of the in-plane σ-bonding. Otherwise, the magnetic exchange between copper ions is negligible in this compound (G = 4.05).

Analogously to Figure 1, Figure 3 shows the X-ray structure of **2**, and two DFT optimized structures, either using the X-ray coordinates or exchanging the NH_2_/OH groups in the distal position as starting point (2 and 2′DFT, respectively). The geometry obtained by DFT calculations reasonably agrees to that reported in the X-ray structure (Figure 3). Nevertheless, due to the absence of neighboring molecules, non-covalent distances are considerably shorter than the experimental ones. The most significant change is the different conformation of the acv side chain, which herein involves the -O(ol) group in additional H-bonds (Figure 3b,c). The possibility of having different arrangements for the tridentate hen chelator has been also analyzed. In Figure 2b, in agreement with X-ray data, an intramolecular (hen)O-H···O6(acv) H-bond is present. Conversely, in Figure 3c the –NH_2_ group of hen points towards the O6-carbonyl group of acyclovir, thus forming a (hen)N–H···O6(acv) instead of (hen)O–H···O6(acv) H-bonding interaction. This latter conformation is 3.2 kcal/mol higher in energy than **2**, hence less likely to occur, according to the experimental findings.

**Figure 4:**
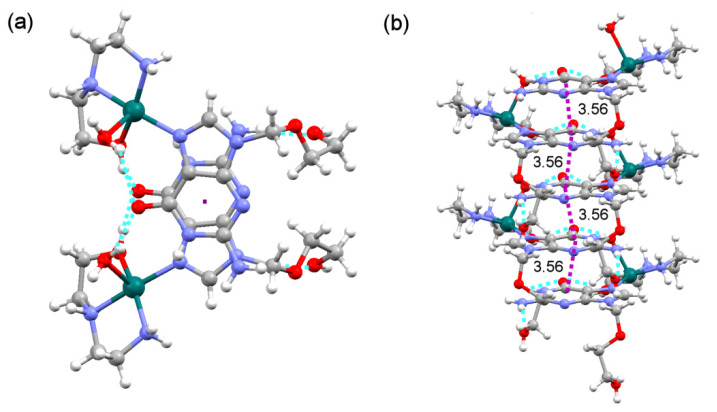
View of the π-stacked complex cations in compound **2** (**a**) in the c axis and (**b**) along the c axis. H-bonds and π,π-stacking interactions are depicted in cyan dotted lines and purple dashed lines, respectively. Nitrate anions are omitted for clarity. Distances given in Å. Atoms colour code: carbon—grey; hydrogen—white; nitrogen—blue; oxygen—red; copper—dark green.

Regarding the crystal structure, rather strong π,π-stacking interactions (interplanar angle α = 0.3°, slipping angles ß or ɤ = 19.7° or 19.6°) among the 6-membered ring of antiparallel acv ligands build multi-stacked ribbons in the c axis (Figure 4). The mean interplanar distance is 3.56 Å, fairly close to that of the stacked nucleobases in a Watson-Crick DNA helix (3.4 Å) [29]. The 3D arrangement is accomplished by further H-bonding interactions that connect the stacked ribbons of complex cations with nitrate anions, thus stabilizing the crystal structure (Table S2.2).

### 2.3. Analysis of the Structural and Theoretical Studies of Compound [Cu(hen)(acv)_2_](ClO_4_)_2_ (***3***)

**Figure 5:**
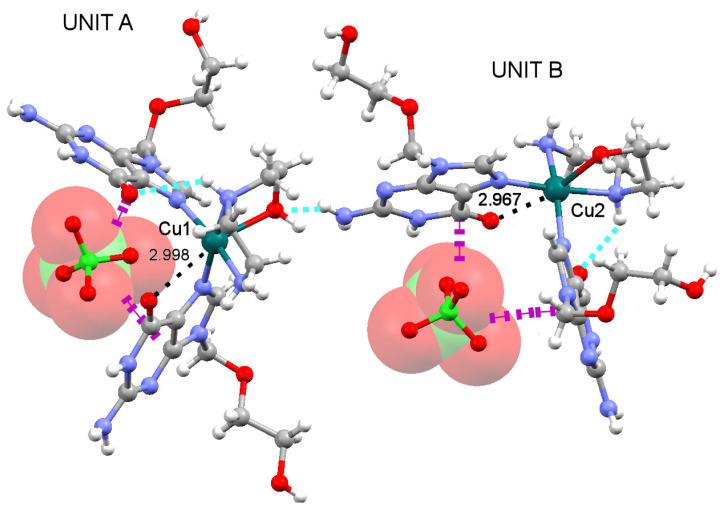
Partial view of the X-ray structure of compound **3** indicating units A and B. Intramolecular H-bonds are depicted as dotted cyan lines. The ancillary Cu···O interactions are represented by dashed black lines with their corresponding distance in Å. The perchlorate anions are represented with transparent CPK style with indication of the anion–π interactions (larger magenta horizontal dashed lines). Atoms colour code: carbon—grey; hydrogen—white; nitrogen—blue; oxygen—red; copper—dark green, chlorine—light green.

In the asymmetric unit of **3**, two crystallographic independent molecules coexist, hereafter called units A and B, corresponding to Cu1 and Cu2 centres respectively (Figure 5). The two non-equivalent copper(II) centres are pentacoordinated, showing a square-based pyramidal geometry, type 4 + 1 (Scheme 1f). The bidentate chelating role of hen drives the *cis*-coordination of both acv ligands in each complex cation. This *cis*-(acv)_2_ coordination has been previously described for platinum(II) compounds [9], and certainly resembles the well described binding of the platinum cationic moiety Pt(NH_3_)^2+^ to DNA guanosine moieties in platinum(II) drugs such as cisplatin [30]. However, it is the first time a copper(II) complex is reported with acyclovir featuring in such conformation. In this context, it is worth mentioning that the *cis*-coordination of two acyclovir ligands to the same Cu(II) centre originates a cleft where the ClO_4_^−^ anion is actually accommodated forming several anion–π interactions with the extended π–surface (Figure 5).

Units A and B exhibit the same molecular recognition pattern. As expected, acyclovir acts in both units as a monodentate ligand via the N7(acv) donor atom. Because the two N-acv donors preferentially occupy basal positions, the tridentate hen chelator is forced to adopt a fac-N_2_+O(distal) conformation, as previously reported in compound **1** (Scheme 1f, Table S3.1). In the structure, only one acv ligand per unit formally assists the Cu-N7(acv) bond by an intramolecular H-bond type (hen)N-H···O6(acv), involving the secondary amino group of hen (Figure 5, Table S3.2). Nonetheless, one might also consider fairly weak (~3 Å) H-bonding interactions type (hen)N-H···O6(acv) for the other acv ligand but involving the primary amine of hen instead. Interestingly, the carbonyl O6-atom of the acv ligand that is not formally involved in an intramolecular H-bond is located at approximately 3 Å from the Cu(II) metal centre, in both units A and B, thus establishing some kind of non-covalent or ancillary Cu···O interaction (Figure 5). These two facts, along with the *cis*-coordination of the acv ligands, certainly contribute to the large dihedral angle observed between the mean basal Cu(II) coordination plane and each of the two guanosine/acv planes per unit (59.04° and 70.66° for unit A, and 53.18° and 77.81° for unit B).

EPR spectra of compound **3** are quite complicated due to the simultaneous presence of contributions from two magnetically independent Cu(II) resonant species (Figure S4.3). The narrow line observed in the high-field region of the Q-band spectrum indicates that both centres have axial symmetry. Furthermore, the perpendicular components of the *g* tensors are close (*g*_⊥_~2.05), which confirms the similar geometry on the equatorial plane and dx^2^ − y^2^ ground state in both Cu(II) ions. The poor resolution of the low-field region of the spectra indicates larger differences between the parallel *g* components (*g*_‖_~2.17–2.22) due to larger differences in the distal Cu-O(hen) bond lengths (Table S3.1). The absence of hyperfine structure implies that extended magnetic exchange is present in this compound. However, considering the thermal evolution of the X-band EPR spectra, it can be concluded that the two Cu(II) subnets are coupled separately, and that the magnitude of the exchange interaction is small but different for each of them.

**Figure 6:**
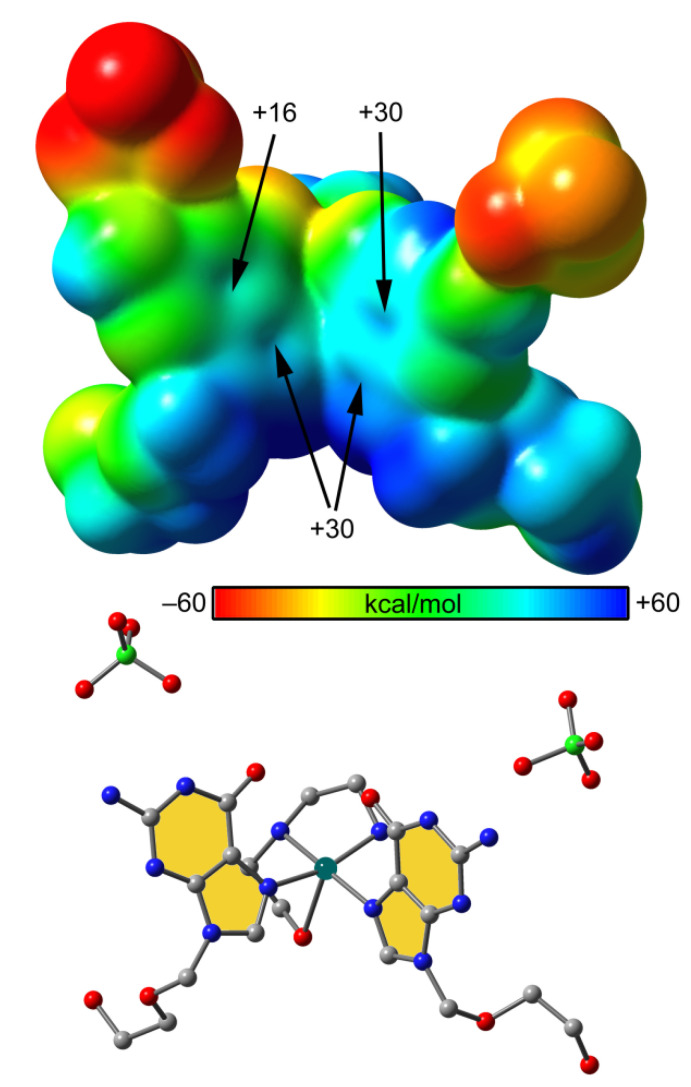
Top: MEP surface plotted onto the 0.001 a.u. isosurface. The values at selected points of the surface are given in kcal/mol. Bottom: Fragment used for the MEP (unit A, compound **3**) at the same orientation used for the MEP surface representation (H-atoms omitted).

In order to evaluate the ability of the purine moiety of acyclovir to interact with anions, unit A was used as a model of the [Cu(acv)_2_(hen)]^2+^ complex to compute the molecular electrostatic potential (MEP) surface. Two counter anions were included in the model with the aim of having a neutral system for the calculation. The results are shown in Figure 6 where it can be observed a positive MEP value over the π-systems, ranging from +16 to +30 kcal/mol, hence supporting the suitability of the anion–π interactions described above.

**Figure 7:**
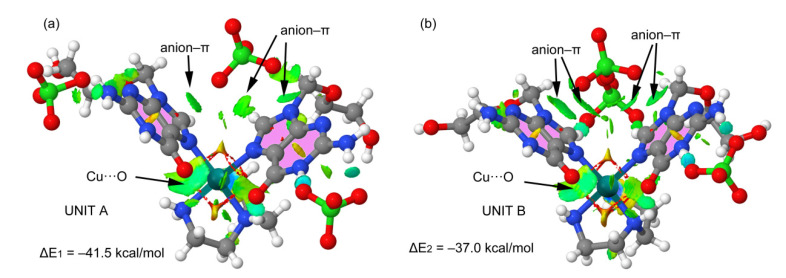
NCI surfaces of the anion–π complexes in units A (**a**) and B (**b**). The gradient cut-off is s = 0.35 au, and the color scale is −0.04 < ρ < 0.04 au. The interactions energies at the B3LYP-D3/def2-SVP level of theory are also indicated. Atoms colour code: carbon—grey; hydrogen—white; nitrogen—blue; oxygen—red; copper—dark green, chlorine—light green.

The anion–π interactions were further analyzed using the Non-Covalent Interactions (NCI) plot index. This index and its associated isosurfaces allow identifying which regions of a supramolecular complex are actually involved in non-covalent interactions and their complementarity. The non-covalent contacts are revealed by reduced density gradient (s) regions at low densities (ρ), which are plotted by using isosurfaces. The isosurfaces are colour coded according to ρ values, where yellow and red correspond to weak and strong repulsive interactions, respectively; and green and blue are used for weak and strong attractive interactions, respectively [31]. The NCIplots for units A and B of compound **3** are given in Figure 7. Green (attractive) and large isosurfaces can be found between the O(perchlorate) atoms and the π-systems of both acv ligands, clearly supporting the presence of anion–π interactions. Moreover, the interaction energies for the anion–π complexes were computed in both units, with their large value (ΔE_1_ = −41.5 and ΔE_2_ = −37.0 Kcal/mol, Figure 7) confirming the relevance of these interactions in the solid state of compound **3**.

**Figure 8:**
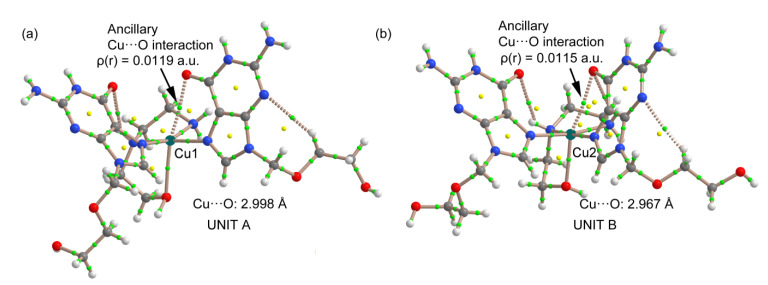
AIM distribution of bond and ring critical points (green and yellow spheres, respectively) and bond paths for units A (**a**) and B (**b**) in compound **3**. Atoms colour code: carbon—grey; hydrogen—white; nitrogen—blue; oxygen—red; copper—dark green, chlorine—light green.

The NCIplot also shows a green isosurface between the carbonyl O(acv) atoms and the Cu(II) centres, characterizing the ancillary Cu···O interactions as non-covalent bonding. In order to confirm the existence of such ancillary Cu···O interactions in both units of compound **3**, the Bader’s quantum theory of “atoms in molecules” (QTAIM) analysis was performed, since the existence of a bond path connecting two atoms provides a universal indicator of bonding between them [32]. Figure 8 shows the presence of a bond critical point (CP) and bond path connecting the carbonyl O6-atom of acv to the Cu(II) atom in both units (represented as dashed bond path), thus confirming the interaction. Moreover, the small value of ρ at the bond CP is a clear indication of the non-covalent nature of the interaction, in agreement with the long Cu···O distance (~3.0 Å) and the previous NCIplot analysis.

## 3. Materials and Methods

### 3.1. Reagents

Inorganic copper salts CuSO_4_·5H_2_O, Cu(NO_3_)_2_·3H_2_O and the rather hygroscopic Cu(ClO_4_)_2_·6H_2_O and acyclovir (acv) were purchased from Merck. N-(2-hydroxyethyl)ethylenediamine (hen) was provided by Alfa Aesar. Since hen is a very viscous liquid at room temperature, in order to improve reproducibility within the synthesis, a methanol mother solution of hen was prepared. To this purpose, 5 mL of hen were dissolved in pure methanol up to a total volume of 250 mL. Hence, a solution of hen 0.2 M is obtained from which 5 mL aliquots contain 1 mmol of hen (MW = 104.15 g/mol; d = 1.029 g/mL). All reagents were used as received without additional purification.

### 3.2. Synthesis

#### 3.2.1. [Cu(hen)_2_]SO_4_ (**1**)

In a Kitasato flask, CuSO_4_·5H_2_O (0.25 mmol) was dissolved in 20 mL methanol. Then 2.5 mL of hen mother solution (0.5 mmol) were slowly added. The reacting mixture instantly showed an intense blue colour, and was allowed stirring for 1 h and a half. The final clear solution was filtered in a crystallization device to remove possible un-reacted materials. The filtered solution was left at room temperature under controlled evaporation of the solvent. After 10 days, suitable crystals for single crystal X-ray diffraction appeared, leading to the structure of compound **1**. Yield ca. 75%. Elemental analysis (%): Calc. for C_8_H_24_CuN_4_O_6_S, C 26.12, H 6.58, N 15.23; Found: C 26.05, H 6.68, N 15.31. FT-IR [KBr, cm^−1^]: ν(OH)_ol_ 3425, ν_as_(NH_2_) 3364 broad, νs(NH_2_) 3247 and 3207, ν(NH) 3127, ν_as_(CH_2_) 2954 and 2940, ν_s_(CH_2_) 2882 and 2847, δ(NH_2_) 1581, δ(NH) 1520, δ(OH)_ol_ 1381, ν_3_(SO_4_^2−^) 1119, ν_1_(SO_4_^2−^) 980, ν_4_(SO_4_^2−^) 618, ν_2_(SO_4_^2−^) 457. The thermogravimetric (TG) curve was divided in five steps. During the TG experiment, a series of 35 FT-IR spectra of the gasses evolved during pyrolysis were recorded, showing H_2_O, CO_2_, MeOH, CO, NH_3_, N-oxide gasses (N_2_O, NO and NO_2_), SCNH and ethylene. A final residue of CuO was expected (950 °C, Calc. 22.62%; Found 22.56%).

#### 3.2.2. [Cu(hen)(acv)(H_2_O)](NO_3_)_2_ (**2**)

CuNO_3_·3H_2_O (1 mmol) was dissolved in 40 mL methanol. Then 5 mL of hen mother solution (1 mmol) were slowly added. The reacting mixture instantly showed an intense blue colour. Afterwards 1 mmol of acv was added, which further intensified the colour. The reacting mixture was stirred for 3 h and then filtered in a crystallization device to remove possible un-reacted materials. The filtered solution was left at room temperature under controlled evaporation of the solvent. After 10 days, colourless needle-like crystals appeared that were identified as acv by FT-IR. Hence, the solution was again filtered to remove undesired nucleation sites. The filtered solution was then placed under diethyl ether diffusion, which worked as anti-solvent. After 8 h, blue needle-like crystals suitable for single crystal X-ray diffraction appeared, leading to the structure of compound **2**. Yield ca. 89%. Elemental analysis (%): Calc. for C_12_H_25_CuN_9_O_11_, C 26.94, H 4.71, N 23.57; Found: C 27.03, H 4.81, N 23.42. FT-IR [KBr, cm^−1^]: ν(OH)_ol_ 3404, ν_as_(NH_2_) 3319, ν_s_(NH_2_) 3252 ν(NH) 3167 and 3122, ν_as_(CH_2_) 2946, ν_s_(CH_2_) 2853, ν(C=O)_acv_ 1664, δ(NH_2_) 1600, δ(NH) 1544 and 1520, δ(OH)_ol_ 1382, ν_as_(C–O–C)_acv_ 1126, ν_3_(NO_3_^−^) 1366, ν_2_(NO_3_^−^) 850. Diffuse reflectance spectrum shows an asymmetric d-d band with λ_max_ at 627 nm (ν_max_ 15,950 cm^−1^). The thermogravimetric curve showed five steps. During the TG experiment, a series of 35 FT-IR spectra of the gasses evolved during pyrolysis were recorded, showing H_2_O, CO_2_, CO, N-oxide gasses (N_2_O, NO and NO_2_) and 1,3-dioxolane. The coordinated water molecule is partially lost during the exposure to air-dry flow prior the TG experiment and the first step of the TG experiment (from 75 to 150 °C). A non-pure final residue of CuO was observed (535 °C, Calc. 14.87%; Found 17.50%).

#### 3.2.3. [Cu(hen)(acv)_2_](ClO_4_)_2_ (**3**)

Cu(ClO_4_)_2_·6H_2_O (1 mmol) was dissolved in 40 mL methanol. Then 5 mL of hen mother solution (1 mmol) were slowly added. The reacting mixture instantly showed an intense blue colour. Afterwards 1 mmol of acv were added, which further intensified the colour. The reacting mixture was stirred for 1 h and then filtered in a crystallization device to remove possible un-reacted materials. The filtered solution was left at room temperature under controlled evaporation of the solvent. After 2 months, in order to promote crystallization, the crystallization device was moved under 2-propanol diffusion, which worked as anti-solvent. After 15 days, multiple parallelepipedal blue crystals suitable for single crystal X-ray diffraction appeared, leading to the structure of compound **3**. Yield ca. 25%. Elemental analysis (%): Calc. for C_20_H_32_Cl_2_CuN_12_O_15_, C 29.47, H 3.96, N 20.62; Found: C 29.17, H 4.02, N 20.57. FT-IR [KBr, cm^−1^]: ν(OH)_ol_ overshadowed, ν_as_(NH_2_) 3396 and 3300, ν_s_(NH_2_) 3241, ν(NH) 3176 and 3129, ν_as_(CH_2_) 2938, ν_s_(CH_2_) 2883, ν(C=O)_acv_ 1695, δ(NH_2_) 1633 and 1584, δ(NH) 1548, δ(OH)_ol_ 1354, ν_as_(C–O–C)_acv_ 1110 broad, ν_3_(ClO_4_^−^) 1089 broad. Diffuse reflectance spectrum shows an asymmetric d-d band with λ_max_ at 600 nm (ν_max_ 16,667 cm^−1^). Thermogravimetric experiments cannot be carried out in compounds containing perchlorate ions due to their explosive character.

### 3.3. Single-Crystal X-ray Diffraction

Measured crystals were prepared under inert conditions immersed in perfluoro-polyether as protecting oil for manipulation. Suitable crystals were mounted on MiTeGen Micromounts^TM^, and these samples were used for data collection. Data were collected with Bruker D8 Venture diffractometer (Bruker-AXS, Billerica, Massachusetts, USA) at 100(2) K. Graphite monochromated MoKα radiation (λ = 0.71073 Å) was used throughout. The data were processed with APEX3 suite [33]. The structures were solved by intrinsic phasing using the ShelXT program [34], which revealed the position of all non-hydrogen atoms. These atoms were refined on F^2^ by a full-matrix least-squares procedure using the anisotropic displacement parameter [35]. All hydrogen atoms were located in difference Fourier maps and included as fixed contributions riding on attached atoms with isotropic thermal displacement parameters 1.2 or 1.5 times those of the respective atom, except for those belonging to the –OH and NH_2_ groups, which were also located in difference Fourier maps. Geometric calculations were carried out with PLATON [36] while Olex2 software was used as a graphical interface [37]. Molecular graphics were generated using Olex2 [37] and Mercury [38]. Crystallographic data for the reported structures have been deposited in the Cambridge Crystallographic Data Centre with numbers 2,062,498–2,062,500. Relevant crystal data are also provided in the Supplementary Materials (S1–S3). Copies of this information could be obtained free of charge on application at http://www.ccdc.cam.ac.uk/products/csd/request (accessed on 10 February 2021).

### 3.4. Other Physical Measurements

Elemental analyses were performed with a Thermo Scientific Flash 2000 (Thermo Fisher Scientific Inc. Waltham, MA, USA). Infrared spectra were recorded by using KBr pellets on a Jasco FT-IR 6300 spectrometer (Jasco Analítica, Madrid, Spain). Thermogravimetric analyses (pyrolysis) of the studied compounds were carried out in air-dry flow (100 mL/min) by a Thermobalance Mettler-Toledo TGA/DSC1 (Mettler-Toledo, Columbus, Ohio, USA), and a series of FT-IR spectra of evolved gasses were recorded for the studied compounds using a coupled FT-IR Nicolet 550 spectrometer (Thermo Fisher Scientific Inc. Waltham, MA, USA). Electronic (diffuse reflectance) spectra were obtained in a Varian Cary-5E spectrophotometer (Agilent Scientific Instruments, Santa Clara, CA, USA) from grinded crystalline sample. Electron paramagnetic resonance (EPR) measurements were performed using a Bruker ELEXSYS E500 spectrometer operating at the X-band. The spectrometer was equipped with a super-high-Q resonator ER-4123-SHQ. Solid samples and toluene solutions were placed in quartz tubes and spectra were recorded at different temperatures between 5 and 300 K with standard Oxford Instruments low temperature devices using typical modulation amplitudes of 1.0 G at a frequency of 100 kHz. The magnetic field was calibrated by a NMR probe and the frequency inside the cavity (~9.4 GHz) was determined with an integrated MW-frequency counter. Room temperature Q-band (~33.9 GHz) spectra were also recorded on solid samples using a Bruker EMX system equipped with an ER-510-QT resonator. Data was collected and processed using the Bruker Xepr suite.

### 3.5. Theoretical Methods

The energetic and geometric features of the complexes included in this study were calculated at the B3LYP-D3/def2-SVP level of theory using the crystallographic coordinates as starting geometries for compounds **1** and **2** and without further optimization for compound **3** (only the positions of the H-atoms were optimized). For the calculations, the GAUSSIAN-16 program has been used [39]. The basis set superposition error for the calculation of interaction energies has been corrected using the counterpoise method [40]. Molecular electrostatic potential (MEP) surfaces have been computed at the same level of theory and represented using the 0.001 a.u. isosurface. The NCI plot index [31] computational tool has been used to characterize non-covalent interactions using the B3LYP-D3/def2-SVP level wavefunction [41,42,43,44,45]. They correspond to both favourable and unfavourable interactions, as differentiated by the sign of the second density Hessian eigenvalue and defined by the isosurface colour. The colour scheme is a red-yellow-green-blue scale with red for ρ + cut (repulsive) and blue for ρ − cut (attractive). Yellow and green isosurfaces correspond to weakly repulsive and attractive interactions, respectively. The QTAIM analysis [46] has been performed using the AIMAll program at the same level of theory [47].

## 4. Conclusions

Guanosine and, by extension, acyclovir preferentially binds metal ions via its N7 donor atom. Although N7 is not the most basic donor in the purine moiety, this atom offers two advantages: (1) it is actually less hindered than the N1 donor, which is more basic but it has the O6-carbonyl group in *ortho* position; and (2) it offers the possibility to reinforce the coordination bond via one H-bonding interaction using the aforementioned carbonyl O6 atom of the guanosine moiety as H-acceptor. For instance, this molecular recognition pattern has been described for the binding of cis-platinum drugs to DNA. In this work, we observe the same trend for the synthetic nucleoside acyclovir, but herein a more versatile ligand has been used to promote the formation of such H-bonding interaction. Thus, the ligand hen exhibits one primary plus one secondary amino group and one terminal O-H group able to work as H-donors. When the flexible hen ligand adopts a *mer*-NO_2_ conformation (i.e., both the (hen)amino and (hen)O-H groups are localized within the basal copper(II) coordination plane) the intramolecular (hen)O-H···O6(acv) H-bond is promoted. This observation is in agreement with the highest polarity of the terminal (hen)O-H group versus that of both N-H groups, thus favouring its implication. On the other hand, when the hen ligand adopts a *fac*-N_2_+O conformation (i.e., the (hen)O-H group is localized at the distal Cu(II) coordination site) there is no actual competition between the referred hen H-donors and the (hen)N-H in the basal coordination place is the one preferred for building the intramolecular H-bonding interaction, type (hen)N-H···O6(acv).

## Data Availability

The data presented in this study are available in this article or supplementary material.

## Supplementary Materials

The following are available online at https://www.mdpi.com/1424-8247/14/3/244/s1, Tables S1.1, S2.1 and S3.1 contain relevant bond lengths [Å] and angles [°] for compounds **1**–**3**, respectively. Tables S1.2, S2.2 and S3.2 contain canonical hydrogen bonds [Å and °] for compounds **1**–**3**, respectively. Figures S4.1, S4.2 and S4.3 show the powder EPR spectra (right: Q-band; Left: X band) of compounds **1**–**3**, respectively.
