# Supplementary material for: Deciphering the H-Bonding Preference on Nucleoside Molecular Recognition through Model Copper(II) Compounds
Source: Pharmaceuticals (Basel). 2021 Mar 9;14(3):244. doi: 10.3390/ph14030244 (PMC7998196; doi:10.3390/ph14030244)
Supplement: Supplementary file 1 [file pharmaceuticals-14-00244-s001.pdf]

pharmaceuticals

Supporting Information

Deciphering the H-bonding preference on nucleoside molecular recognition through model copper(II) compounds

Inmaculada Velo-Gala [1], Miquel Barceló-Oliver [2], Diego M. Gil [3], Josefa M. González-Pérez [4], Alfonso Castiñeiras [5] and Alicia Domínguez-Martín [4,*]

[1] Faculdade de Engenharia da Universidade do Porto. Rua Dr. Roberto Frias, 4200-465 Porto; invega@fe.up.pt
[2] Department of Chemistry, Universitat de les Illes Balears, Crta de Valldemossa km 7.5, 07122 Palma de Mallorca (Baleares), Spain; miquel.barcelo@uib.es
[3] INBIOFAL (CONICET-UNT). Instituto de Química Orgánica. Facultad de Bioquímica, Química y Farmacia. Universidad Nacional de Tucumán. Ayacucho 471. T4000INI. San Miguel de Tucumán. Argentina; dmgil@fbqf.unt.edu.ar
[4] Department of Inorganic Chemistry, Faculty of Pharmacy, University of Granada, 18071 Granada, Spain; jmgp@ugr.es
[5] Department of Inorganic Chemistry, Faculty of Pharmacy, University of Santiago de Compostela, 15782 Santiago de Compostela, Spain; alfonso.castineiras@usc.es
* Author Correspondence: adominguez@ugr.es

CONTENT

S1. Relevant crystallographic information regarding [Cu(hen)$_2$]SO$_4$ (1)

Table S1.1. *Bond lengths [Å] and angles [°] for compound **1**.*

Table S1.2. *Hydrogen bonds for compound **1** [Å and °].*

Table S1.3. *Crystal data and structure refinement for compound **1**.*

S2. Relevant crystallographic information regarding [Cu(hen)(acv)(H$_2$O)](NO$_3$)$_2$ (2)

Table S2.1. *Bond lengths [Å] and angles [°] for compound **2**.*

Table S2.2. *Hydrogen bonds for compound **2** [Å and °].*

Table S2.3. *Crystal data and structure refinement for compound **2**.*

S3. Relevant crystallographic information regarding [Cu(hen)(acv)$_2$)](ClO$_4$)$_2$ (3)

Table S3.1. *Bond lengths [Å] and angles [°] for compound **3**.*

Table S3.2. *Hydrogen bonds for compound **3** [Å and °].*

Table S3.3. *Crystal data and structure refinement for compound **3**.*

S4. EPR and magnetic properties

Figure S4.1. *Experimental and simulated powder EPR spectra of compound **1**. Right: Q-band, RT. Left: X band, 5 K.*

Figure S4.2. *Experimental and simulated powder EPR spectra of compound **2**. Right: Q-band, RT. Left: X band, 5 K.*

Figure S4.3. *Experimental powder EPR spectra of compound **3**. Right: Q-band, at different temperatures. Left: X band, 5 K.*

S1. Relevant crystallographic information regarding [Cu(hen)$_2$]SO_4 (1)

Table S1.1. *Bond lengths [Å] and angles [°] for compound **1**.*

Cu(1)-N(1)	2.044(3)
Cu(1)-N(4)	2.028(3)
Cu(1)-O(7)	2.410(2)
Cu(1)-N(11)	2.035(3)
Cu(1)-N(14)	2.016(3)
Cu(1)-O(24)	2.879(3)
O(7)-Cu(1)-O(24)	169.00(8)
N(11)-Cu(1)-N(1)	173.47(12)

Table S1.2. *Hydrogen bonds for compound **1** [Å and °].*

D-H...A	d(D-H)	d(H...A)	d(D...A)	<(DHA
N(1)-H(1)...O(23)#1	1.00	1.92	2.901(4)	166.2
N(4)-H(4A)...O(22)	0.91	2.29	3.184(4)	166.3
N(4)-H(4B)...O(25)#2	0.91	2.08	2.915(4)	152.0
O(7)-H(7)...O(22)#3	0.89	1.83	2.703(3)	166.8
N(11)-H(11)...O(25)	1.00	1.89	2.889(4)	172.0
N(14)-H(14A)...O(24)#1	0.91	2.24	2.963(4)	135.5
N(14)-H(14B)...O(23)#3	0.91	2.08	2.958(4)	163.1
O(17)-H(17)...O(23)#2	0.84	1.96	2.794(4)	173.3

Symmetry transformations used to generate equivalent atoms:
#1 x-1/2,-y+1/2,-z+1 #2 -x+2,y+1/2,-z+1/2 #3 x-1,y,z

Table S1.3. *Crystal data and structure refinement for compound* ***1****.*

Empirical formula	C8 H24 Cu N4 O6 S	
Formula weight	367.91	
Temperature	99.8 K	
Wavelength	0.71073 Å	
Crystal system	Orthorhombic	
Space group	$P2_12_12_1$	
Unit cell dimensions	a = 8.5032(4) Å	α= 90°.
	b = 8.9206(5) Å	ß= 90°.
	c = 18.7305(13) Å	γ = 90°.
Volume	1420.78(14) $Å^3$	
Z	4	
Density (calculated)	1.720 Mg/m^3	
Absorption coefficient	1.715 mm^{-1}	
F(000)	772	
Crystal size	0.12 x 0.1 x 0.08 mm^3	
Theta range for data collection	2.175 to 27.488°.	
Index ranges	-10<=h<=10, -11<=k<=11, -23<=l<=24	
Reflections collected	9795	
Independent reflections	3239 [R(int) = 0.0593]	
Completeness to theta = 25.242°	99.8 %	
Absorption correction	Semi-empirical from equivalents	
Max. and min. transmission	0.7456 and 0.5784	
Refinement method	Full-matrix least-squares on F^2	
Data / restraints / parameters	3239 / 0 / 182	
Goodness-of-fit on F^2	1.038	
Final R indices [I>2sigma(I)]	R1 = 0.0333, wR2 = 0.0689	
R indices (all data)	R1 = 0.0383, wR2 = 0.0709	
Absolute structure parameter	0.021(9)	
Extinction coefficient	n/a	
Largest diff. peak and hole	1.139 and -0.715 e.$Å^{-3}$	

S2. Relevant crystallographic information regarding [Cu(hen)(acv)(H_2O)](NO_3)$_2$ (2)

Table S2.1. *Bond lengths [Å] and angles [°] for compound* **2**.

Cu(1)-N(1)	1.985(5)
Cu(1)-O(1)	2.241(4)
Cu(1)-O(4)	1.985(4)
Cu(1)-N(7)	1994(5)
Cu(1)-N(27)	2.010(5)
N(1)-Cu(1)-N(27)	173.0(2)
O(4)-Cu(1)-N(7)	161.4(2)

Table S2.2. *Hydrogen bonds for compound* **2** *[Å and °].*

D-H...A	d(D-H)	d(H...A)	d(D...A)	<(DHA
N(1)-H(1)...O(14A)	1.0	1.88	2.820(7)	155.5
O(1)-H(1A)...O(12A)	0.91	2.32	2.863(8)	118.4
O(1)-H(1B)...O(26)#1	0.91	1.99	2.846(6)	157.1
O(4)-H(4)...O(26)	0.88	1.99	2.648(6)	131
O(4)-H(4)...O(26)#2	0.88	2.51	3.062(6)	122
N(7)-H(7A)...O(44)#1	0.91	2.01	2.895(7)	164.8
N(7)-H(7B)...O(43)#2	0.91	2.47	3.113(7)	128.0
N(7)-H(7B)...O(43)#3	0.91	2.43	3.173(7)	139.1
N(21)-H(21)...O(44)	0.88	2.02	2.874(7)	164.7
N(22)-H(22A)...O(34)#1	0.88	2.00	2.827(8)	155.3
N(22)-H(22B)...O(43)	0.88	2.30	3.080(8)	148.1
O(34)-H(34)...O(13A)#4	0.84	2.17.	2.779(8)	129.7
O(34)-H(34)...O(13B)#4	0.84	1.94	2.667(13)	144.3

Symmetry transformations used to generate equivalent atoms:

#1 x,-y+1/2,z-1/2 #2 x,-y+1/2,z+1/2 #3 -x+1,y-1/2,-z+1/2 #4 x+1,y,z+1

Table S2.3. *Crystal data and structure refinement for compound* **2**.

Empirical formula	C12 H25 Cu N9 O11	
Formula weight	534.95	
Temperature	100.0 K	
Wavelength	0.71073 Å	
Crystal system	Monoclinic	
Space group	$P2_1/c$	
Unit cell dimensions	a = 13.2050(8) Å	α= 90°.
	b = 23.2386(14) Å	ß= 91.460(3)°.
	c = 7.0783(3) Å	γ = 90°.
Volume	2171.4(2) $Å^3$	
Z	4	
Density (calculated)	1.636 Mg/m^3	
Absorption coefficient	1.081 mm^{-1}	
F(000)	1108	
Crystal size	0.1 x 0.08 x 0.08 mm^3	
Theta range for data collection	2.335 to 25.026°.	
Index ranges	-15<=h<=15, -25<=k<=27, -7<=l<=8	
Reflections collected	15347	
Independent reflections	3761 [R(int) = 0.0680]	
Completeness to theta = 25.242°	95.6 %	
Absorption correction	Semi-empirical from equivalents	
Max. and min. transmission	0.7456 and 0.6309	
Refinement method	Full-matrix least-squares on F^2	
Data / restraints / parameters	3761 / 15 / 291	
Goodness-of-fit on F^2	1.048	
Final R indices [I>2sigma(I)]	R1 = 0.0676, wR2 = 0.1654	
R indices (all data)	R1 = 0.0964, wR2 = 0.1831	
Extinction coefficient	n/a	
Largest diff. peak and hole	2.008 and -1.242 e.$Å^{-3}$	

S3. Relevant crystallographic information regarding [Cu(hen)(acv)$_2$)](ClO$_4$)$_2$ (3)

Table S3.1. *Bond lengths [Å] and angles [°] for compound* ***3****.*

Cu(2)-N(9)	1.974(4)
Cu(2)-N(87)	1.995(4)
Cu(2)-N(67)	1.997(4)
Cu(2)-N(2)	2.037(4)
Cu(2)-O(7)	2.476(4)
Cu(1)-N(27)	1.995(4)
Cu(1)-N(3)	2.008(4)
Cu(1)-N(47)	2.010(4)
Cu(1)-N(1)	2.032(4)
Cu(1)-O(5)	2.306(3)
N(9)-Cu(2)-N(87)	163.07(17)
N(67)-Cu(2)-N(2)	175.45(17)
N(27)-Cu(1)-N(3)	169.32(17)
N(47)-Cu(1)-N(1)	174.24(15)

Table S3.2. *Hydrogen bonds for compound* ***3*** *[Å and °].*

D-H...A	d(D-H)	d(H...A)	d(D...A)	<(DHA
N(9)-H(9A)...N(23)#1	0.88	2.27	3.101(6)	156.7
N(9)-H(9B)...O(31)#1	0.78	2.56	3.027(6)	120.1
N(9)-H(9B)...O(34)#1	0.78	2.18	2.938(6)	163.9
O(94)-H(94)...O(78)#2	0.89	1.84	2.720(8)	167.6
N(2)-H(2)...O(86)	0.88	2.27	2.982(5)	137.7
N(2)-H(2)...O(32)#3	0.88	2.66	3.316(7)	132.1
N(2)-H(2)...O(36)#3	0.88	2.52	3.198(8)	133.9
O(7)-H(7)...O(12)#4	1.04	2.39	3.045(7)	120.1
O(7)-H(7)...O(42)#4	1.04	2.11	3.023(6)	145.4
N(61)-H(61)...O(86)#3	0.88	2.05	2.830(5)	147.0
N(62)-H(62A)...O(86)#3	0.79	2.16	2.892(5)	153.8
N(62)-H(62A)...O(36)	0.79	2.57	3.058(8)	121.1
N(62)-H(62B)...O(5)	0.82	2.27	3.005(5)	150.4

N(81)-H(81)...O(23)#3	0.88	2.04	2.885(5)	161.7
N(82)-H(82A)...O(24)#3	0.78	2.18	2.960(6)	173.1
N(82)-H(82B)...O(46)#2	0.95	1.98	2.879(5)	156.5
N(82)-H(82B)...O(46)#5	0.95	2.52	3.148(5)	123.6
O(5)-H(5)...O(94)#6	0.91	1.90	2.739(5)	152.9
O(34)-H(34)...N(43)#1	0.82	2.09	2.889(6)	164.0
N(1)-H(1)...O(26)	0.86	2.46	3.087(5)	129.9
N(1)-H(1)...O(13)	0.86	2.30	2.972(6)	135.4
N(3)-H(3A)...N(83)#6	0.81	2.38	3.083(5)	146.2
N(3)-H(3A)...O(46)	0.81	2.44	2.982(5)	125.5
N(3)-H(3B)...O(91)#6	0.81	2.44	3.112(5)	141.6
N(3)-H(3B)...O(94)#6	0.81	2.30	2.982(6)	142.5
N(21)-H(21)...O(43)	0.88	2.05	2.928(6)	177.5
N(22)-H(22A)...O(44)	0.82	2.14	2.950(6)	167.8
N(22)-H(22B)...O(66)#7	0.82	2.14	2.925(5)	160.8
N(41)-H(41)...O(26)#8	0.88	2.07	2.851(5)	148.0
N(42)-H(42A)...O(26)#8	0.98	2.05	2.935(6)	149.0
N(42)-H(42A)...O(12)#8	0.98	2.54	3.075(7)	114.1
N(42)-H(42B)...O(7)#9	0.88	2.09	2.960(6)	167.7

Symmetry transformations used to generate equivalent atoms:

#1 -x+1/2,y+1/2,-z+1/2 #2 -x+3/2,y+1/2,-z+1/2

#3 -x+1,-y+1,-z #4 -x+1,-y+1,-z+1 #5 x+1/2,-y+1/2,z-1/2

#6 -x+3/2,y-1/2,-z+1/2 #7 -x+1/2,y-1/2,-z+1/2

#8 -x+1,-y,-z+1 #9 x,y-1,z

Table S3.3. *Crystal data and structure refinement for compound* ***3****.*

Empirical formula	C20 H34 Cl2 Cu N12 O15	
Formula weight	817.03	
Temperature	100(2) K	
Wavelength	0.71073 A	
Crystal system, space group	Monoclinic, P 21/n	
Unit cell dimensions	a = 18.512(2) A	α = 90 deg.
	b = 18.570(2) A	ß = 98.035(5) deg.
	c = 19.072(2) A	ɣ = 90 deg.
Volume	6492.0(12) A^3	
Z, Calculated density	8, 1.672 Mg/m^3	
Absorption coefficient	0.925 mm^-1	
F(000)	3368	
Crystal size	0.137 x 0.038 x 0.025 mm	
Theta range for data collection	2.222 to 26.733 deg.	
Limiting indices	-23<=h<=21, -23<=k<=23, -24<=l<=24	
Reflections collected / unique	144006 / 13778 [R(int) = 0.0754]	
Completeness to theta = 25.242	99.9 %	
Absorption correction	Semi-empirical from equivalents	
Max. and min. transmission	1.000 and 0.925	
Refinement method	Full-matrix least-squares on F^2	
Data / restraints / parameters	13778 / 0 / 941	
Goodness-of-fit on F^2	1.035	
Final R indices [I>2sigma(I)]	R1 = 0.0726, wR2 = 0.1901	
R indices (all data)	R1 = 0.1021, wR2 = 0.2134	
Extinction coefficient	n/a	
Largest diff. peak and hole	2.276 and -1.254 e.A^-	

S4. Relevant EPR and magnetic properties

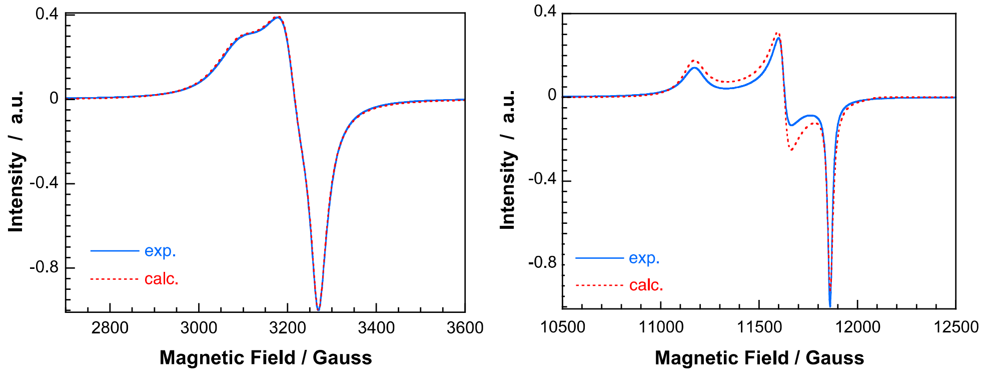

Figure S4.1. *Experimental and simulated powder EPR spectra of compound* **1**. *Right: Q-band, RT. Left: X band, 5 K.* The principal components of the g tensor have been estimated by comparison of the experimental Q-band spectra with those obtained by a computer simulation program working at the second order of the perturbation theory. The calculated *g* values are: $g_1 = 2.186$; $g_2 = 2.094$; $g_3 = 2.053$ ($\langle g \rangle = 2.111$).

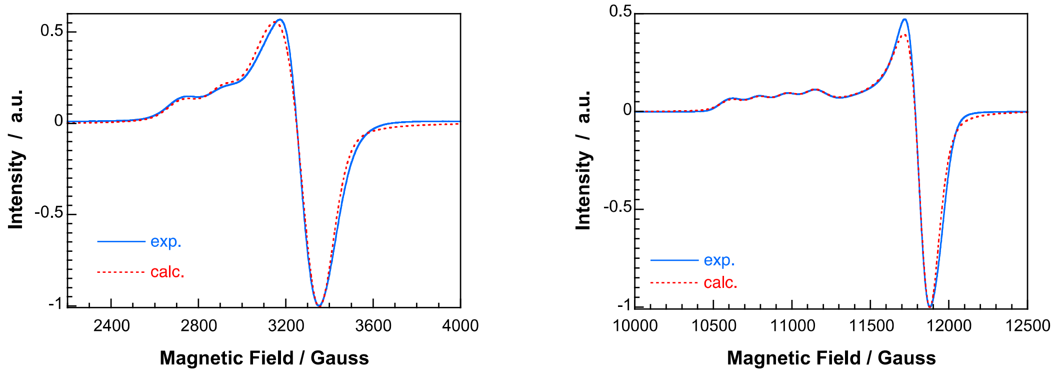

Figure S4.2. *Experimental and simulated powder EPR spectra of compound* **2**. *Right: Q-band, RT. Left: X band, 5 K.*

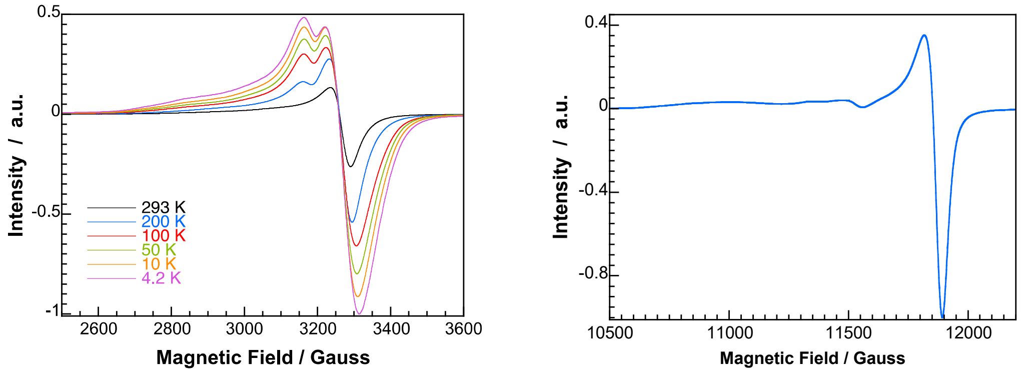

Figure S4.3. *Experimental powder EPR spectra of compound **3**. Right: Q-band, at different temperatures. Left: X band, 5 K.*
